# Acceptance of COVID-19 Vaccines in India: A Systematic Review and Meta-Analysis

**DOI:** 10.3390/vaccines11050964

**Published:** 2023-05-09

**Authors:** Gunjan Kumar, Samikshya Jena, Niher Tabassum Snigdha, Sakeenabi Basha, Jayaraj Kodangattil Narayanan, Alexander Maniangat Luke

**Affiliations:** 1 Department of Public Health Dentistry, Kalinga Institute of Dental Sciences, KIIT Deemed to Be University, Patia, Bhubaneswar 751024, Odisha, India; 2 Paediatric Dentistry Unit, School of Dental Sciences, Universiti Sains Malaysia, Health Campus, Kota Bharu 16150, Kelantan, Malaysia; 3 Department of Community Dentistry, Faculty of Dentistry, Taif University, P.O. Box 11099, Taif 21944, Saudi Arabia; 4 Medical and Dental Sciences Department, College of Dentistry, Ajman University, Ajman P.O. Box 346, United Arab Emirates; 5 Department of Clinical Science, College of Dentistry, Ajman University, Al-Jurf, Ajman P.O. Box 346, United Arab Emirates; 6 Centre of Medical and Bio-allied Health Sciences Research, Ajman University, Al-Jurf, Ajman P.O. Box 346, United Arab Emirates

**Keywords:** vaccine acceptance, vaccine hesitancy, COVID-19 vaccine, India, SARS-CoV-2, willingness

## Abstract

This systematic review and meta-analysis sought to evaluate the acceptability levels for COVID vaccine(s) in various states in India. Published articles in PubMed/Scopus/Cochrane/DOAJ/the Web of Science that focused on assessing COVID-19 vaccine hesitation/vaccine acceptance using a survey/questionnaire were included. After extensive research, 524 records were found, and after screening on the basis of eligibility criteria, only 23 papers were added to this review. Increased vaccine assumption percentage (>70%) among the population was found in two surveys nationwide (92.8%) and in Delhi (79.5%). For pooled estimates of COVID-19 vaccine acceptance and heterogeneity, twenty-three studies (23) consisting of 39,567 individuals reported for acceptance of COVID 19 vaccine in India.. Out of these, 26,028 individuals accepted the COVID-19 vaccine, giving a pooled estimate of 62.6% (95% CI: 55.6–69.4) with considerable heterogeneity (χ^2^ = 3397.3, *p* < 0.0001; I^2^ = 99.40%). The results of this study give a brief insight into the percentage acceptance and hesitancy among the Indian population regarding COVID-19 vaccine immunisation. Future research and vaccine education initiatives can be steered by the findings of this work as a starting point.

## 1. Introduction

The World Health Organization (WHO) revealed that COVID-19, a global pandemic, was brought on by the Severe Acute Respiratory Syndrome Corona Virus 2 (SARSCoV2) on 11 March 2020 [1]. Many nations resorted to a national or regional lockdown to halt the spread of the pandemic, and various behavioural treatments were advocated [2].

Lockdowns, fatalities, and economic slowdowns have been felt by towns and countries around the world since the coronavirus disease (COVID-19) first appeared [3]. The extreme contagiousness of this illness and the predicted mortality rate, which was substantially greater than that of the seasonal flu, posed significant disruptions and risks to day-to-day living [4]. Nations increased the development and testing of novel medications and vaccinations at the same time as they attempted to adapt the use of currently available medical interventions for COVID-19 treatment [5]. Global efforts were made to combat this highly infectious disease, in response to which vaccines were developed globally [6].

The Indian Ministry of Health worked with AstraZeneca to develop immunisation and sell it for a reasonable price. AstraZeneca and the Serum Institute of India worked together to mass-manufacture COVISHIELD in India [7]. COVAXIN was the second vaccine produced by the Indian biotech firm Bharat Biotech. Following that, on 16 January 2021, it began the world's largest vaccination drive with COVISHIELD and COVAXIN. Vaccination first started among front-line worriers, which consisted of healthcare personnel, sanitation workers, law enforcement officers, volunteers, emergency medical technicians, and many others who courageously supported the state and central governing bodies in the hour of the nation’s emergency [8]. Both vaccines were available free of cost throughout the country, in various government centres of all levels for all the citizens of the nation. Both types of vaccines were available differently according to the locations so as to distribute the vaccine to the majority of the population and reach the remotest parts of the country [9]. This was a marvelous step taken by the government of India to encourage the people to avail themselves to the immunisation drive. Until the end of 2021, 1.1% of the population received two doses of the vaccine, while approximately 120 million received only one [10]. According to the WHO, 1.45 billion doses of the vaccine were expected to be delivered by the end of May 2022 in India in order to achieve herd immunity. Initially, it was a challenge to achieve the goal, but later on with increasing knowledge and awareness regarding the importance and effectiveness of the COVID-19 vaccine among the people, India could successfully immunise around 2 billion people by the start of the year 2023 [11]. 

India had a minimal population response rate, and 6.5% of its vaccines were wasted [12]. Equivalent considerations regarding receiving the COVID-19 vaccination have been seen in surveys conducted in the UK, USA, and France [13]. Despite the enormous efforts put forth by both government and non-government bodies to develop effective immunisation against COVID-19, a significant barrier could be attributed to vaccine apprehension against the authorised and anticipated COVID-19 immunisation [14,15]. The people’s hesitation and reluctance to receive immunisations, despite the implementation of vaccinations that have dramatically decreased severe COVID-19 symptoms and mortality, is a barrier in India to advancing worldwide health [16]. Vaccine ambivalence, defined by the WHO as a delay in adoption, a hesitancy to embrace, or a denial of vaccines in the face of accessible immunisation facilities, is as common in India as it is across the rest of the globe [17]. Several research studies conducted in India observed that the primary causes of vaccine reluctance in the context of childhood immunisation were cited as vaccine safety, rumour and controversy regarding the harmful effects following vaccination, a deficient understanding of the advantages of immunisations, expenses, and conventional belief systems [8,17,18]. The overall acceptance rate of the COVID-19 vaccine was satisfactory. The majority of the health workers accepted COVID-19 vaccination, understanding the need of the situation and severity of the spread of the disease. Among the general population, few considered that taking the vaccine against COVID-19 was important to protect themselves from the fatal disease.

To recognise the extent of this issue, this systematic review and meta-analysis sought to evaluate the acceptability rates for COVID-19 vaccine(s) in various states in India. It can serve as a starting point for future surveys into the data involved in the geographical and intellectual disparities that contribute to COVID-19 vaccination reluctance.

## 2. Materials and Methods

### 2.1. Study Protocol and Registration

This systematic review was administered in accordance with PRISMA specifications. Articles were reposted from internet database sources on 19 September 2022. This review was recorded in PROSPERO with the registration code CRD42022353572.

### 2.2. Research Questions

The research questions considered for this systematic review included the following. What are the factors that positively or negatively influence decision-making on COVID-19 vaccine acceptance? What is the proportion of the Indian population that would agree or deny taking the COVID-19 vaccine? What are the different percentages of vaccine acceptance for COVID-19 among different states of India? What is the extent of variation in the vaccine’s approval rate for the COVID-19 among several age groups and genders?

### 2.3. Data Sources

Relevant articles meeting the inclusion criteria were found using keywords that included coronavirus terms (coronavirus OR corona-virus OR COVID OR COVID-19 OR COVID-2019 OR severe acute respiratory syndrome coronavirus OR severe acute respiratory syndrome coronavirus 2 OR 2019-nCoV” OR SARS-CoV-2 OR 2019-nCoV) and vaccine acceptance/rejection terms. The Boolean operators used to compile the keywords were “OR” and “AND,” as shown in Table 1.

### 2.4. Search Strategies

A comprehensive data search was conducted in electronic online databases for original full-text research papers based on the keywords issued until December 2022. The total studies attained from each database are illustrated in Table 1. The full-text research was conducted online in five journal databases using Medical Subject Heading keywords. The articles assessed were from PubMed, Scopus, Cochrane, DOAJ, and the Web of Science.

### 2.5. Selection Criteria

The selection parameters were:

1. Peer-reviewed articles that have been published and are indexed in PubMed, Scopus, Cochrane, DOAJ, or the Web of Science.
2. Researches conducted in India.
3. The paper’s main objective was to test COVID-19 vaccination uptake or resistance.
4. The study should be published in English language.

The parameters for elimination were:

1. Manuscripts that have not been published.
2. Studies that did not assess COVID-19 vaccination acceptance or reluctance.
3. A publication language other than English.

### 2.6. Type of Studies Included

Observational studies (including case-control studies) will be included to assess the prevalence of acceptance. Qualitative studies will be included to assess the barriers to hesitancy and factors for assessing the COVID-19 vaccine for the Indian population.

### 2.7. Data Extraction (Selection and Coding)

The primary review authors (G.K. and S.J.) independently screened the titles and abstracts of the literature search results together with an assigned co-screener (N.T.S.), and (J.K.N.) and (A.M.L.) provided supervisory oversight and validated the selection process. Disagreements were resolved through discussions, and the final decision was made by either S.B. or A.M.L. The full-text articles were then independently screened for inclusion and further processing. As needed, corresponding authors were contacted to provide the missing information. A distinctively customised Excel sheet was fabricated to register the data obtained from the studies. The data extraction sheet was independently used by the two reviewers to capture the following data items for each of the articles included in this review:

1. General information about the article, such as the author’s name, title, and setting (Indian states).
2. The study’s aim or objectives, the study’s design, and the sampling
3. Participant characteristics, including mean age and gender distribution.
4. Outcome data and results, including the unit of analysis, outcome (reported or not), the definition of the outcome, factors, and barriers reported.
5. Limitations of the study.

### 2.8. Quality Assessment

The studies were approved for eligibility and omission based on their titles, abstracts, and inclusion and exclusion criteria. After complete screening, full-text studies were evaluated individually, and their superiority was analysed. To assess the quality of the included research, the JBI critical assessment checklist was used. This checklist has nine items to assess (i) a sample frame that addresses the specific audience; (ii) appropriate selecting methods; (iii) sufficient sample sizes; (iv) study participant and setting characterisations; (v) enough statistical investigations; (vi) uses appropriate techniques for the aforementioned parameters; (vii) uses valid measurements for all the study subjects; (viii) the use of appropriate statistical analysis; and (ix) an adequate response rate. Answers such as yes, no, unclear, or not applicable are assigned to each item. The “yes” response was marked with green, the “no” response was marked with red, and the “unclear” response was marked with yellow (Table 2).

### 2.9. Statistical Analysis

The study results were synthesised by a quantitative summary of the evidence, where the pooled prevalence estimates were utilised as the quantitative summary. Data analysis was performed using R Software version 4.2.2, and forest plots were employed to visually summarise the effect of each study along with its confidence interval and to estimate the pooled effect size. To account for expected heterogeneity, a random-effects model was applied in this meta-analysis. Additionally, a meta-regression analysis is also performed, which is depicted in Table 3. The I^2^ statistic was employed to assess the variability among the studies, with high values indicating strong heterogeneity. Given the selection of nationally representative samples and primary studies with relatively smaller sample sizes, high heterogeneity was anticipated. Hence, a subgroup analysis was conducted based on the gender and residence that might differ significantly across the studies. Additionally, funnel plot asymmetry was used to detect publication bias, while Begg’s correlation test and Egger’s regression test were utilised to quantitatively assess the possibility of publication bias. A *p*-value of <0.05 was considered statistically significant.

## 3. Results

### 3.1. Study Selection Results

**Figure 1:**
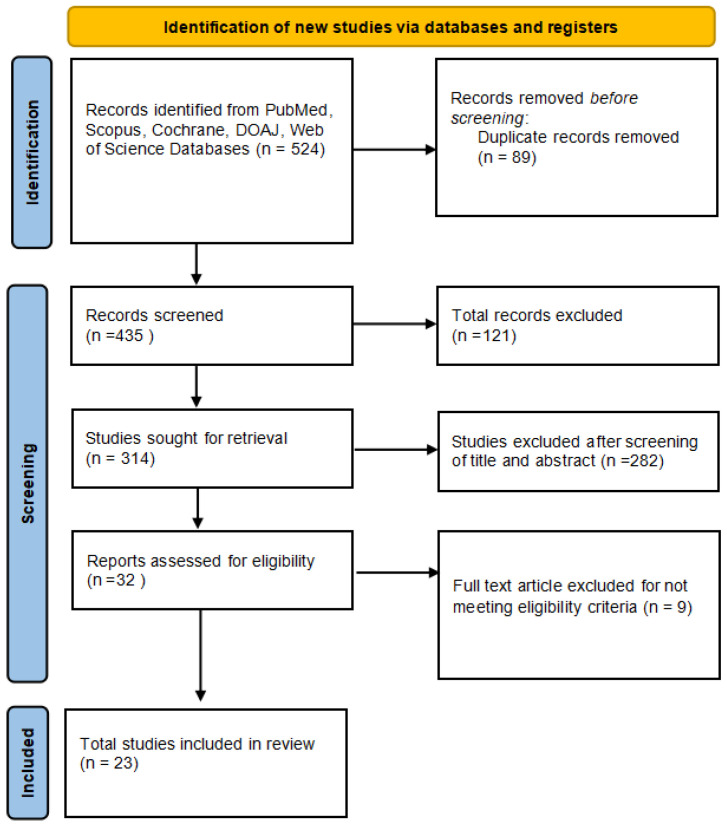
PRISMA flowchart.

A total of 524 records were identified, including 203 articles found in PubMed, 108 in Scopus, 79 in Cochrane, 83 in DOAJ, and 51 in the Web of Science. A total of 89 articles were duplicated, and they were excluded during the screening of the articles. The MeSH terms were used in all searches. After reading the titles and abstracts of the articles, 282 studies were eliminated. Out of the 32 remaining full-text articles, 9 studies did not meet the inclusion criteria. A total of 23 articles on vaccine acceptance in the Indian population were included in this review. Figure 1 depicts the selection criteria it follows the PRISMA guidelines. PRISMA Checklist 2020 is depicted in Supplementary Materials File S1.

### 3.2. Study Feature

The fundamental features of the incorporated articles are summarised in Table 4. The incorporated articles were printed in a good, reputed journal indexed in the Web of Science, Scopus, and PubMed. The majority of the studies (n–4) were published in the Journal of Education and Health Promotion, two in the journal Vaccine, and the remaining studies all had one publication in the following journals: *PloS ONE*, *Journal of Indian Academy of Oral Medicine and Radiology*, *Journal of Pharmacy And Bioallied Sciences*, *Tzu Chi Medical Journal*, *Journal of Family Medicine and Primary Care*, *Roczniki Panstwowego Zakladu Higieny*, *BMC Health Services Research*, *Vacunas*, *Brain*, *Behavior*, *and Immunity—Health*, *Journal of preventive medicine and hygiene*, *Vaccine: X*, *Epidemiology and Infection*, *Indian Journal of Public Health*, *Human Vaccines and Immunotherapeutics*, *Korean Journal of Family Medicine*, *and Asia-Pacific Journal of Public Health*.

### 3.3. *Characteristics of the Papers Included*

A total of 23 published articles were analysed in this review. These articles constituted studies on COVID-19 vaccine approval across India. The highest number of studies were performed nationwide (8), followed by Delhi (3) and Tamil Nadu (2). The dates for survey distribution ranged from July 2020 to February 2022.

The largest sample size (N = 12093) was found in the survey conducted by Sharma P et al. in Delhi, while the smallest sample size (N = 163) was found in the survey conducted in Imphal, Manipur, by Gupta A et al. among pregnant women. Out of the twenty-three studies, eleven were on the general population; ten surveys were on healthcare professionals; one was on transgender people; and one was on pregnant women. The survey was most commonly conducted in the months of February and March.

### 3.4. *Rates of COVID-19 Vaccine Acceptance*

Table 5 displays the results of COVID-19 vaccine acceptance rates and factors that positively or negatively influence the decision-making on COVID-19 vaccine acceptance from various studies included in this review that are divided by different Indian states.

The highest vaccine acceptance percentage (>70%) among the population was found in two articles conducted nationwide (92.8%) and in Delhi (79.5%). On the other side, the lowest vaccine acceptance rate (<60%) among the general public was established in four studies: nationwide (50%), Tamil Nadu (46%), West Bengal (44.33%), and Kashmir (33%).

For the ten studies conducted on healthcare workers, two surveys described an acceptance rate below 60%, which were conducted in Rishikesh (53.4%) and Chandigarh (54.6%). In the studies, a higher rate (>70%) of approval for the COVID-19 vaccine was administered in Uttarakhand (90%), Kashmir (81.5%), and nationwide (73%).

One study was conducted among the transgender community in Tamil Nadu, with a higher response rate (76%) for COVID-19 vaccine acceptance. One survey was carried out among pregnant women in Imphal, Manipur, which reported a lower rate (22.1%) of acceptance for the COVID-19 vaccination. The male gender and the 18-24 age group were associated with significantly higher rates of COVID-19 vaccine acceptance.

Factors positively influencing the acceptance of COVID-19 vaccination are extensive public knowledge about COVID-19, where 70% of the population that had better knowledge about the vaccines [35], and a good attitude towards it. The likelihood that a participant might acknowledge vaccine acceptance increased with their perception of their risk of suffering from COVID-19, their belief that the vaccine would protect them from COVID-19, and their belief that the vaccine would not cause any negative adverse consequences. The single factor that appears to slow the SARS-CoV-2 transmission is vaccination.

Factors negatively influencing acceptance of the COVID-19 vaccination are lower levels of schooling, lower incomes, and underemployment. Everyone’s concern about the vaccine, la dearth of knowledge about the novel COVID 19 vaccine, anti-vaccination beliefs and views, anxiety and worries, concerns about the vaccine’s effectiveness, a concerning level of ignorance, and negligible factual understanding of the COVID-19 outbreak and its accompanying vaccination effort. Most of the pregnant women were reluctant to receive the COVID-19 immunisation as they were unaware of the advantages of COVID-19 vaccination.

**Figure 2:**
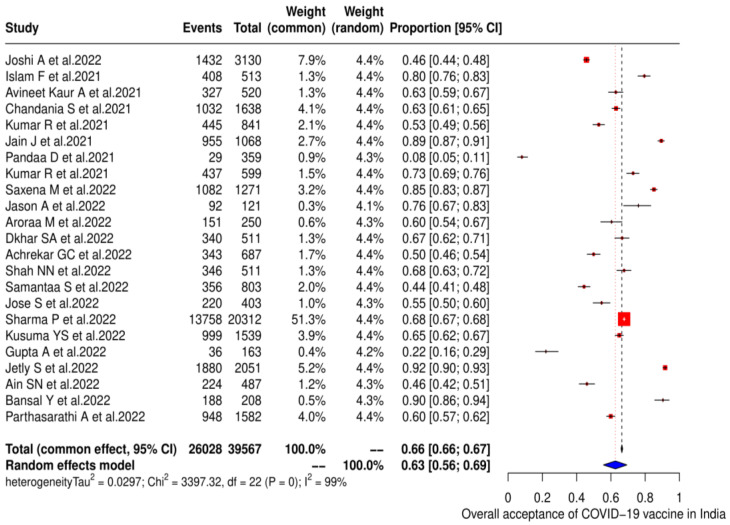
The overall pooled acceptance of the COVID-19 vaccine in India, as shown in the form of a forest plot [19,20,21,22,23,24,25,26,27,28,29,30,31,32,33,34,35,36,37,38,39,40,41].

For pooled estimates of COVID-19 vaccine acceptance and heterogeneity, twenty-three studies (23) consisting of 39,567 individuals reported the proportion of vaccine acceptance in India. Out of these, 26,028 individuals accepted to take the COVID-19 vaccine, providing a pooled estimate of 62.6% (95% CI: 55.6–69.4) with considerable heterogeneity (χ^2^ = 3397.3, *p* < 0.0001; I^2^ = 99.40%) (Figure 2).

**Figure 3:**
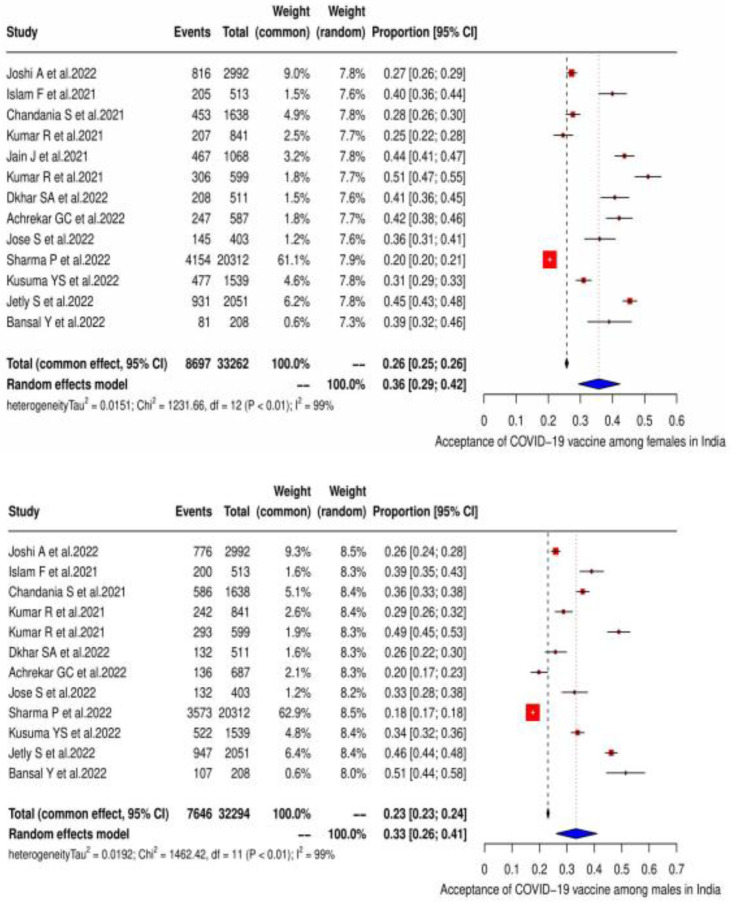
Acceptance of the COVID-19 vaccine in India according to gender, as shown in the form of a forest plot [19,20,21,22,23,24,25,26,27,28,29,30,31,32,33,34,35,36,37,38,39,40,41].

As per the subgroup analysis, the acceptance of the COVID-19 vaccine was found highest among females at 35.7% (95% CI: 29.40–42.3), with varied heterogeneity (χ^2^ = 1232, *p* < 0.0001; I^2^ = 99.0%), as compared with males at 33.30% (95% CI: 26.10–41.0) with heterogeneity of (χ^2^ = 1462.4, *p* < 0.001; I^2^ = 99.20%) (Figure 3 ).

**Figure 4:**
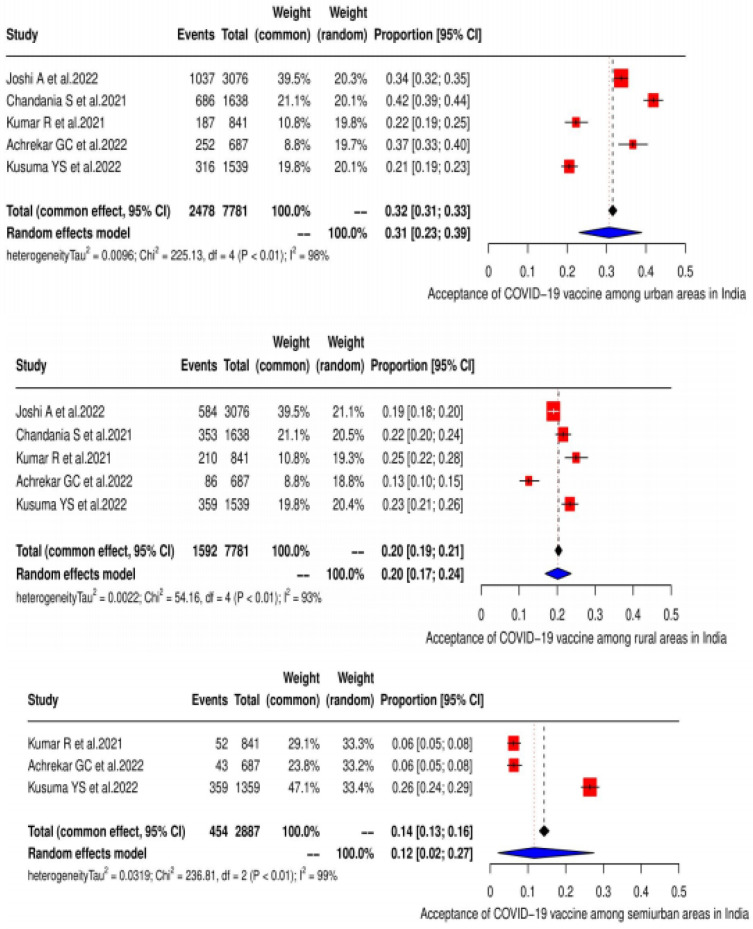
Acceptance of the COVID-19 vaccine in India according to residence, as shown in the form of a forest plot [19,20,21,22,23,24,25,26,27,28,29,30,31,32,33,34,35,36,37,38,39,40,41].

As per the subgroup analysis, the highest acceptance of the COVID-19 vaccine was found in urban areas at 30.7% (95% CI: 23.0–39.0), with varied heterogeneity (χ^2^ = 225, *p* < 0.0001; I^2^ = 98.20%), followed by rural at 20.14% (95% CI: 16.8–23.7) and semiurban at 11.7% (95% CI; 2.10–27.50) (Figure 4), respectively. A detailed description is given in Table 6.

**Figure 5:**
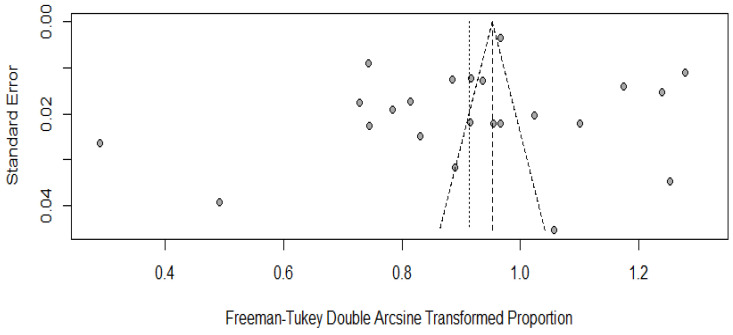
A funnel plot for estimating publication bias.

For estimating the publication bias, we plotted the funnel plot along with Begg’s correlation test, in which a *p*-value of <0.05 was considered significant. In the present study, the *p*-value for Begg’s correlation test comes out to be around 0.4921 (Figure 5).

## 4. Discussion

COVID-19 changed the dynamics of the world. The emergence of COVID-19 created havoc throughout the world [42]. International and national authorities acted swiftly to stop the spread of the disease to as many individuals as possible. Notwithstanding the high death rate observed globally, efforts were undertaken to address the issue [43]. Healthcare workers, front-line workers, scientists, and government and non-government bodies worked relentlessly to provide all the possible facilities to the people suffering from this novel disease. After all the efforts, many countries were able to manufacture their own vaccine against the deadly disease [44]. Because India is a densely populated country, two vaccines were introduced and manufactured in India to meet demand, which were first made freely available to front-line and healthcare workers before being made freely available to the broader public. The recurrence of some contagious diseases, such as measles and pertussis epidemics, illustrates the long-standing theme of vaccine refusal, which poses a severe concern for world welfare. Unexpected advances were made in creating effective and efficient COVID-19 vaccinations in a limited period of time. In spite of the facilities, many people showed hesitancy to accept the vaccine [45]. However, the universal attempt to contain the current epidemic with its deleterious healthcare and economic repercussions might be limited by the public’s disinclination to acquire COVID-19 immunisation [46]. Therefore, assessments of vaccine acceptability levels can indeed be useful in planning the activities and treatment procedures required to uplift public understanding and encourage individuals about the security and benefits of vaccinations, which in turn will assist to curb viral transmission and lessen the adverse outcomes of this unexpected outbreak [47]. An assessment of perceptions and acceptability levels for the COVID-19 vaccine can aid in the launch of much-needed promotional efforts to increase confidence in medical authorities [48].

In this systematic review, comprehensive data were attained, large variability of vaccine acceptance was established, and the reasons for acceptance and hesitancy towards vaccination were evaluated. When a survey of the general population was conducted, many reasons were given for people’s reluctance to receive the COVID-19 vaccine. Joshi A. et al. identified limited economic resources and a lack of literacy as important factors of vaccine reluctance. Those with lower levels of schooling, wage stagnation, and aged 25 to 54 had a higher likelihood of denying the COVID-19 vaccine. Concerns and postponements about the safety of immunisation were the primary reasons for fostering scepticism about the acceptance of immunisation for COVID-19 [19]. In another study by Chandania S et al., 25% of people were either oblivious to immunisations or unsure about receiving them, and 10% said they would not accept the vaccine. Nearly 70% of the populace expressed worries about vaccinations. Considering the vastness of India’s demography, even a small percentage of those who are bothered about achieving immunisation may result in millions of individuals not acquiring the disease [22]. Panda D et al. conducted a study in Odisha and discovered that the majority of participants agreed or accepted that both adults and infants could receive the COVID-19 vaccine safely. Substantial differences were discovered across all the groups in the following areas: the development of greater resistance after the disease rather than through vaccination; efficacy in preventing illness; security of children; availability of government-mandated vaccination; and public health protection following government instructions [25]. The residents residing in West Bengal showed a concerning lack of medical information and a lack of understanding of the COVID-19 pandemic and its associated vaccination campaign [33]. In the study by Sharma P et al., among the population residing in Delhi, most of them were hesitant about the acceptance of the COVID-19 vaccine. The main causes were a lack of knowledge about COVID-19 immunisations and concerns about vaccine efficacy and long-term efficacy [35]. In their survey, Kusuma Y. et al. discovered that the elderly generation has a lower psychological vulnerability to acquiring COVID-19, a lower perceived threat of COVID-19, poor self-defense against COVID-19, and a lack of awareness and non-use of the Aarogya SetuApp as major determinants of vaccine reluctance [36].

Vaccinations were first made available for healthcare workers. A study was conducted among healthcare workers nationwide, which revealed that 84.1% of them accepted the vaccine, as they trusted the initiative by the government of India and the data from previous research [26]. In another study, studies were conducted on healthcare workers, and more than 50% of healthcare workers in Delhi were acquiescent to the vaccine, and 72% felt that immunisation should first be provided to front-line workers [20]. Kaur A. et al. assessed the acceptance of the COVID-19 vaccine, and some participants (45.5% dental and 48.4% medical) expressed worry about unanticipated vaccination side effects. Those who participated in COVID-19 tasks showed greater readiness to get immunised. The readiness of respondents to receive the vaccine increased when they gave the vaccine greater emphasis than natural resistance [21]. Particular antivaccination behaviour and views, anxiety and phobia worries, an insufficient understanding, and concerns about the vaccine’s efficacy were among the causes of vaccine reluctance found among the healthcare professionals of Rishikesh in the study by Kumar R et al. [23]. The greatest vaccination acceptance rate, at 89.8%, was among the medical staff, as reported by Saxena et al. Few were reluctant to get vaccinated due to a variety of factors, including fear of severe adverse symptoms, vaccination effectiveness, incomplete or incorrect data, scepticism of public health authorities, economic considerations, and the notion that they had already developed resistance [27]. In a survey among healthcare workers in Gurugram, nursing personnel were more likely than other healthcare providers to be immunised against COVID-19. The majority of healthcare workers (44%) expressed concern about how quickly vaccines were being developed [29]. Shah NN conducted a survey among healthcare professionals in Kashmir and reported that being unmarried was strongly linked to a higher incidence of vaccine reluctance. The three biggest significant drivers of vaccine opinions were doubts regarding the vaccine’s effectiveness, unanticipated issues in youngsters, and potential upcoming harmful vaccine impacts [32]. In the study by Jose S. et al. among healthcare workers in North India, most people (62%) expressed apprehension about the vaccine’s reliability, including potential risks, manufacturing assurance, and vaccine effectiveness. Uncertainty about the vaccine’s effectiveness was a key indicator of healthcare providers’ reluctance to administer the vaccine [34].

Only one study was conducted on the transgender group, in which 76% of transgender people were immunised, and the adoption and extent of immunisation against COVID-19 were elevated. With very few changes to the ongoing strategies, Bharat could attain complete immunisation against COVID-19 [28]. According to Gupta et al.’s survey of pregnant women in Imphal, Manipur, the most important determinants of vaccine approval were a disagreement that vaccines were dangerous during pregnancy and an acceptance that vaccines were beneficial to the infant^.^ Almost four out of every five pregnant women were reluctant to receive immunisation against COVID-19. The advantages of the COVID-19 vaccine should indeed be explained to them and supported by them [37]. The results of this study give a brief insight into the percentages of acceptance and hesitancy among the Indian population regarding COVID-19 vaccine immunisation. Strict COVID-19 guidelines were formulated by the Government of India for individuals travelling within the country and internationally. Vaccination was made mandatory for those who had to travel, irrespective of the mode of travel. Apart from vaccination, the traveller had to test negative and take proper precautionary measures to be eligible for the trip [10].

According to the recent data obtained, 227,128,569 people have received booster doses in India [49]. Still, there is a very significant population that needs to be vaccinated completely, that is, a first dose, second dose, and booster dose. So, future research and vaccine education initiatives can be guided by the findings of this work as a starting point for inspiration.

### Strengths and Limitations

This study has several strengths. To the best of our knowledge, this is the first comprehensive systematic review and meta-analysis on vaccine acceptance in India. We included well-known, high-standard databases for our search strategy. We searched many databases and found possibly all the published studies related to the acceptance of the COVID-19 vaccine in India. A major limitation was associated with the disparate perspectives used to demonstrate readiness to acquire the COVID-19 vaccine in various types of studies. All the studies included in this research had cross-sectional study designs. The heterogeneity of the data could be explained by the fact that the sample size varied in each study, ranging from a minimum of 121 to a maximum of 20312.

## 5. Conclusions

Large variability of acceptance of the COVID-19 vaccine was published in a different part of the country, India. In this study, the male sex demonstrated higher acceptability of COVID-19 vaccination, supporting the notion that demographic factors played a significant role in the positive trends in vaccine acceptance. Pregnant women demonstrated the least willingness. Only one study was conducted for the vulnerable group, which was the LGBTQ community, which indicated that further studies should be conducted to assess their knowledge, attitude, and acceptance of the COVID-19 vaccine. Therefore, the Indian Government should appreciate vaccine hesitation that exists across India. Aiming to widen the gap, rising hospitalisation and the negative consequences of COVID-19 in unvaccinated people are identified as significant dangers to the nation’s financial and societal sustainability. Developing trust in healthcare systems and vaccination programmes is an important predictor for the community in order to have a successful and painless immunisation for all. More public awareness programmes should be implemented, particularly in rural and underdeveloped areas where people have a widespread misconception about the disease and vaccination [50]. Proper vaccination education may help people change their minds about the vaccination drive.

To address the broader issue of COVID-19 vaccination resistance, governments, healthcare strategy experts, and communication channels, particularly in the digital networking sector, should collaborate. It is suggested to elevate community reliance on COVID-19 immunisation by propagating prompt and condensed messaging via definitive sources that hold up the certainty and potency of the vaccines that are already on the merchandise [51].

## Figures and Tables

**Table 1:** Sources of information and search strategies.

PubMed	(“COVID-19” **OR** ”coronavirus” **OR** ”corona-virus” **OR** ”COVID” **OR** ”COVID-2019” **OR** ”severe acute respiratory syndrome coronavirus” **OR** ”severe acute respiratory syndrome coronavirus 2” **OR** “2019-nCoV” **OR** “SARS-CoV-2” **OR** “2019-nCoV”) **AND** (“Acceptance” **OR** “Hesitancy” **OR** “Willingness”) **AND** (“in”) **AND** (“India”)	203
Scopus	(“COVID-19” **OR** ”coronavirus” **OR** ”corona-virus” **OR** ”COVID” **OR** ”COVID-2019” **OR** ”severe acute respiratory syndrome coronavirus” **OR** ”severe acute respiratory syndrome coronavirus 2” **OR** “2019-nCoV” **OR** “SARS-CoV-2” **OR** “2019-nCoV”) **AND** (“Acceptance” **OR** “Hesitancy” **OR** “Willingness”) **AND** (“in”) **AND** (“India”)	108
Cochrane	(“COVID-19” **OR** ”coronavirus” **OR** ”corona-virus” **OR** ”COVID” **OR** ”COVID-2019” **OR** ”severe acute respiratory syndrome coronavirus” **OR** ”severe acute respiratory syndrome coronavirus 2” **OR** “2019-nCoV” **OR** “SARS-CoV-2” **OR** “2019-nCoV”) **AND** (“Acceptance” **OR** “Hesitancy” **OR** “Willingness”) **AND** (“in”) **AND** (“India”)	79
DOAJ	(“COVID-19” **OR** ”coronavirus” **OR** ”corona-virus” **OR** ”COVID” **OR** ”COVID-2019” **OR** ”severe acute respiratory syndrome coronavirus” **OR** ”severe acute respiratory syndrome coronavirus 2” **OR** “2019-nCoV” **OR** “SARS-CoV-2” **OR** “2019-nCoV”) **AND** (“Acceptance” **OR** “Hesitancy” **OR** “Willingness”) **AND** (“in”) **AND** (“India”)	83
Web of Science	(“COVID-19” **OR** ”coronavirus” **OR** ”corona-virus” **OR** ”COVID” **OR** ”COVID-2019” **OR** ”severe acute respiratory syndrome coronavirus” **OR** ”severe acute respiratory syndrome coronavirus 2” **OR** “2019-nCoV” **OR** “SARS-CoV-2” **OR** “2019-nCoV”) **AND** (“Acceptance” **OR** “Hesitancy” **OR** “Willingness”) **AND** (“in”) **AND** (“India”)	51
Total		524

**Table 2:** JBI checklist for cross-sectional studies.

Study	Q1	Q2	Q3	Q4	Q5	Q6	Q7	Q8	Q9
Joshi A et al. [19]	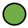	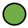	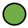	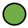	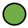	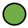	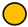	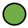	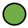
Islam F et al. [20]	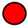	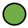	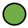	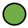	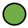	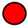	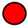	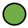	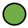
Kaur A et al. [21]	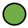	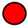	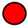	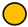	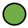	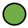	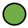	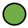	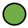
Chandania S et al. [22]	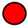	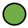	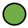	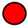	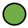	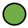	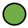	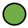	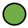
Kumar R et al. [23]	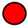	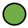	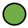	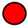	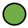	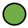	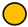	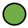	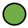
Jain J et al. [24]	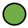	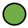	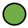	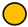	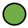	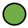	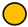	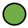	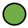
Panda D et al. [25]	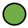	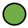	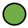	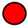	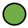	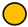	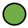	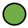	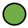
Kumar R et al. [26]	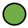	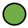	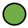	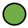	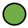	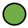	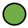	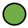	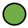
Saxena M et al. [27]	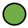	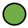	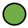	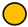	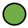	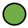	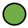	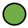	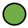
Alden SJ et al. [28]	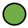	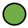	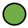	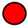	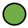	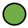	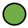	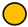	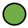
Arora M et al. [29]	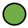	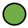	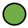	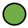	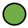	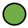	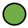	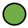	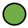
Dkhar SA et al. [30]	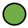	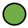	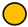	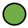	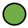	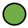	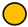	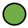	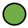
Achrekar GC et al. [31]	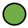	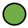	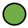	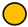	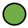	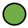	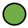	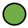	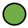
Shah NN et al. [32]	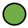	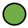	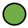	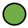	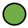	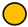	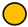	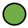	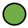
Samanta S et al. [33]	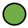	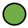	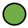	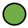	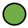	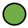	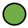	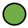	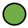
Jose S et al. [34]	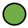	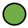	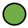	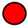	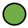	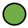	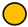	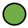	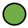
Sharma P et al. [35]	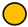	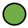	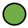	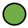	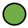	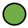	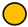	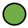	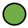
Kusuma Y et al. [36]	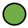	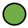	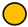	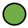	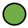	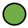	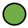	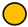	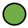
Gupta A et al. [37]	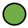	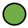	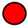	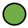	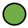	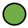	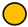	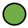	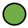
Jetly S et al. [38]	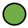	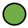	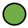	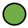	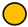	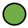	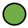	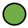	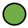
Ain SN et al. [39]	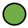	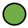	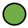	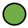	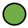	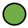	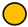	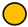	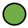
Bansal Y et al. [40]	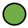	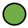	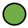	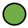	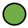	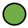	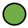	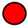	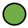
Parthasarathi A et al. [41]	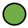	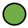	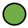	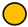	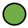	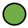	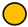	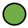	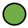

**Table 3:** A univariate meta-regression analysis of various variables regarding COVID-19 vaccine acceptance in India.

Variables	Β Coefficient	95% Confidence Interval		*p*-Values
		Lower	Upper	
Year of publication	−0.31	−0.87	0.24	0.2646
Gender				
Male	−0.71	−1.00	−0.42	<0.0001
Female	−0.60	−0.82	−0.37	<0.0001
Residence				
Urban	−0.83	−1.22	−0.43	<0.0001
Rural	−1.39	−1.66	−1.10	<0.0001
Semiurban	−2.14	−3.25	−1.03	0.0002

**Table 4:** Studies included in the systematic review.

SL. NO.	Author	Journal	Year of Publication
1.	Joshi A et al. [19]	*PloS ONE*	2022
2.	Islam F et al. [20]	*Journal of Family Medicine and Primary Care*	2022
3.	Kaur A et al. [21]	*Roczniki Panstwowego Zakladu Higieny*	2022
4.	Chandania S et al. [22]	*Brain, Behavior, and Immunity—Health*	2022
5.	Kumar R et al. [23]	*Journal of Education and Health Promotion*	2022
6.	Jain J et al. [24]	*Epidemiology and Infection*	2022
7.	Panda D et al. [25]	*Human Vaccines and Immunotherapeutics*	2022
8.	Kumar R et al. [26]	*Korean Journal of Family Medicine*	2021
9.	Saxena M et al. [27]	*Journal of Indian Academy of Oral Medicine and Radiology*	2021
10.	Alden SJ et al. [28]	*Journal of Pharmacy And Bioallied Sciences*	2022
11.	Arora M et al. [29]	*Tzu Chi Medical Journal*	2022
12.	Dkhar SA et al. [30]	*Journal of Education and Health Promotion*	2021
13.	Achrekar GC et al. [31]	*Vaccines*	2022
14.	Shah NN et al. [32]	*Journal of Education and Health Promotion*	2021
15.	Samanta S et al. [33]	*Vacunas*	2022
16.	Jose S et al. [34]	*Journal of preventive medicine and Hygiene*	2022
17.	Sharma P et al. [35]	*Cureus*	2021
18.	Kusuma Y et al. [36]	*Vaccines*	2022
19.	Gupta A et al. [37]	*Indian Journal of Public Health*	2021
20.	Jetly S et al. [38]	*Asia-Pacific Journal of Public Health*	2021
21.	Ain SN et al. [39]	*Journal of Education and Health Promotion*	2021
22.	Bansal Y et al. [40]	*Journal of Family Medicine and Primary Care*	2022
23.	Parthasarathi A et al. [41]	*Vaccines*	2022

**Table 5:** COVID-19 acceptance rate divided by the included studies.

Study Reference	Study Location	Target Population	Total Sample (N)	Questionnaire Used	Response Recorded for Acceptance of Vaccine	Result	Conclusion
[19]	Tamil Nadu	Population- based	3130	CO-VIN-CAP survey	Yes	Males were more likely to appreciate the COVID-19 vaccine (54%) than were people in the 18–24 age group (62%). People aged around 18 and 25 years old were resistant to the COVID-19 vaccine. Those with lower levels of schooling, lower incomes, and underemployment were more likely to reject the vaccine.	The primary causes of reluctance to the COVID-19 immunisation (63%) were worries and uncertainties regarding its efficacy.
[20]	Delhi	Residents	513	Validated semi-structured	Yes	Amongst those who participated in the survey, 79.5% stated they would get the vaccine, 8.8% said they would not, and 11.7% had not made up their minds.	The research aided in understanding the proportion of individuals who were apprehensive about receiving the vaccine, as well as everyone’s worries about the shot.
[21]	Punjab and Uttar Pradesh	Dental and medical professionals	520	Validated self-structured reliability, 0.82	Somewhat/totally agree	From the survey, 63% of individuals demonstrated a readiness to receive the vaccine, while 65% had a favourable view of immunisation. Dental students (45.5%) and medical students (48.4%) expressed worry about unanticipated vaccination side effects. The number of individuals who participated in COVID-19 tasks were 3.45 times more likely to receive immunisation.	The survey’s conclusions demonstrated that the respondents’ acceptability of and attitudes towards the COVID-19 vaccine were less than ideal because of a number of reasons that attributed to the participants’ reluctance to receive the vaccine.
[22]	Nationwide	Population- based	1638	Validated self-structured	Yes	Over a fifth of people (20.63%) were apparently uninformed of the immunisations or unsure of whether they would receive the vaccine (27%), and 10% declined to get the vaccine. Nearly 70% of the populace had reservations about vaccines.	Considering the vastness of India‘s demographic, even a tiny percentage of those who are concerned about getting the vaccine will result in thousands of people not getting it.
[23]	Rishikesh	Healthcare professionals	841	Validated self-structured	Yes	A large proportion of individuals (53.4%) were eager to receive the vaccine, 27.2% were unsure, and 19.4% had no intention of receiving the shot.	Certain antivaccination beliefs and views, anxiety and worries, a dearth of knowledge, and concerns about the vaccine’s effectiveness were among the causes of vaccine reluctance.
[24]	Nationwide	Medical students	1068	Self-structured	Yes	Hesitation towards vaccination was seen in 10.6% of subjects. Individuals who were apprehensive towards vaccinations were more inclined to get their knowledge from digital networking than from their medical school professors.	Medical students’ reluctance to get the COVID-19 vaccination may be significantly diminished by specific information efforts, administrative control of vaccine trials, general disclosure of security and effectiveness results, and measures to foster confidence.
[25]	Odisha	Population- based	359	Self-structured	Strongly agree/agree	Most participants firmly agreed or agreed that both the elderly and infants can get the COVID-19 vaccine without harm.	Security and understanding were reported to be the main obstacles to the COVID-19 vaccine. However, the population of Odisha, India, has a good attitude towards the COVID-19 vaccine.
[26]	Nationwide	Healthcare workers, including physicians, residents, and nurses	599	Validated self-structured	Yes	Approximately 73% of HCWs agreed to receive the vaccines, while 10.85% (n = 65) objected and 16.2% (n = 96) required more time to make up their minds. Questions about vaccine effectiveness and security, antivaccination attitudes and attitudes, individual preference, and a reluctance to receive a vaccine before others were the causes of vaccine hesitation.	Most healthcare workers volunteered to receive COVID-19 vaccinations when they became accessible.
[27]	Nationwide	Healthcare professionals	1271	Validated self-structured	Totally agree/ Agree	The greatest proportion of individuals who have never received a vaccination is seen in the age category of 18 to 45 years, at 14.7%. A total of 13.5% of men and 13.1% of women report not having received a vaccination. The highest vaccination rate, at 89.8%, was among medical staff.	The public had an excellent degree of vaccination acceptability for COVID-19 vaccinations, at around 63%, and their alarming amount of vaccine hesitation, at 27%, was influenced by socioeconomic, societal, and cultural reasons.
[28]	Tamil Nadu	Transgender	121	Self-structured	Yes	The vaccination rate was about 76%, and both the acceptability and accessibility of the vaccine were significant.	Public knowledge of COVID-19 has expanded. India may attain complete vaccination with hardly any investment under the existing regulations.
[29]	Gurugram	Healthcare professionals	250	Validated self-structured reliability, 0.85	Somewhat/completely agree	The majority of medical experts (60.4%) said they would accept vaccination as soon as it is made accessible. Compared to various medical practitioners, nurses were more likely to acknowledge COVID-19 immunisation.	The general approach regarding immunisation was favourable; however, there are many people who have particular reservations about the COVID-19 vaccine.
[30]	Nationwide	An allopathic or alternative system of medicine doctors	511	Validated self-structured	Definitely or probably willing	A total of 340 people (66.53%) answered that they would either definitely or probably embrace COVID-19 vaccination. The likelihood that a participant might acknowledge the vaccine increased with their perception of their risk of catching COVID-19, their belief that the vaccine would protect them from COVID-19, and their belief that the vaccine would not cause any negative adverse consequences.	Before the vaccine is released, a targeted and strengthened lobbying programme for clinicians is required.
[31]	Nationwide	Population- based	687	Psychometric valid tools, vaccine confidence index	Yes	In the study, 44.1% of the individuals did not want to accept the booster injection.	The survey’s results show the necessity for scientific proof initiatives to encourage vaccine acceptance, especially in difficult-to-reach groups in emerging nations.
[32]	Kashmir	Healthcare workers	511	Validated self-structured reliability, 0.93	Yes	A total of 67.7% of healthcare workers expressed a readiness to receive the COVID-19 vaccine if it became accessible. A total of 22.7% of the participants were undecided, and 9.59% said they would not be ready to take the COVID-19 vaccine.	COVID-19 vaccination reluctance was exhibited in a substantial percentage of healthcare workers.
[33]	West Bengal	Population- based	803	Questionnaires from previous studies	Yes	Among the respondents, 12.08% disagreed that getting immunised against COVID-19 was vital, but the remaining participants were divided: 44.33% of participants said they would get the vaccine as soon as it became accessible, while 39.60% said they would wait until thereafter.	Amidst the individuals’ high vaccination attitudes, the results showed a concerning level of ignorance and negligible factual understanding of the COVID-19 outbreak and its accompanying vaccination effort.
[34]	Chandigarh	Nurses	403	Validated self-structured reliability, 0.78	Definitely yes	The large percentage (54.6%) of the 403 research study participants said they would certainly get immunised against COVID-19, although 7% of them said they would not be receptive to immunisation.	In an effort to overcome the pervasive scepticism about the efficacy and effectiveness of vaccinations and achieve adequate protection to build immune systems, communication, management, and vaccination readiness campaigns need to be developed.
[35]	Delhi	Population- based	12093	Self-structured	Yes	The subjects’ approval percentage for the vaccine was 67.7%, with 6031 (43.8%) receiving just one dosage and 7727 (56.2%) receiving two. Among the respondents, just 35.6% said they would get their kids vaccinated.	Individuals who had never received a vaccine did not accept it. Additionally, a significant rate of subsequent immunisation delays was noted.
[36]	Delhi	Population- based	1539	Pre-tested	Accept	In total, 64.9% of participants said they would take the vaccine, 17.4% said they were unsure, and 17.7% rejected receiving the vaccine.	The SARS-CoV-2 vaccination will be welcomed by 2/3rds of Delhi’s lower socioeconomic classes. The elderly, low perceived vulnerability, reduced reported perception intensity, and low self-efficacy to defend oneself from COVID-19 were all connected with vaccine hesitation.
[37]	Imphal, Manipur	Pregnant women	163	Self-structured from previous studies	Accept	Vaccination reluctance was evaluated in 127 (77.9%) respondents.	Most pregnant women were reluctant to receive COVID-19 immunisation. The advantages of the COVID-19 vaccine should be explained to and supported among expecting mothers.
[38]	Nationwide	Population- based	2051	Self-structured	Accept	The majority of the individuals, 1880 (92.8%), acknowledged the COVID-19 vaccine, whereas 146 (7.2%) showed some reluctance. The younger generation had the greatest rates of COVID-19 vaccination acceptability (70.4%) and hesitation (79.3%) compared to the elderly.	The single factor that appears to slow the SARS-CoV-2 transmission is vaccination. The finding suggests that in order for the wider populace to make an intelligent choice, a vaccine educational programme must be made available.
[39]	Kashmir	Population- based	487	Self-structured	Yes	Only 14% of the participants were absolutely resistant to getting the COVID-19 vaccine, and 40% were not sure if they would.	The COVID-19 vaccination-related vaccine reluctance should be reduced by targeted measures.
[40]	Uttarakhand	Medical students	208	Self-structured	Yes	Around 10% of the respondents stated that they were unsure about receiving the subsequent dosage of the vaccine.	Identifying and addressing potential difficulties in reducing stress connected to vaccinations is mandatory to reduce hesitation.
[41]	Nationwide	Population- based	1582	Self-structured	Yes	Around 9% of the respondents rejected vaccination and 30.8% were reluctant.	For certain populations who are more likely to refuse the vaccine, the fundamental social and demographic factors must be handled and outreach campaigns must be developed.

**Table 6:** A gender- and residence-wise subgroup meta-analysis of COVID-19 vaccine acceptance in India.

Categories	Number of Studies	Events	Total Observations	% (95% CI)	Q	I^2^	*p*-Value
**Overall COVID-19 vaccine acceptance**							
**Pooled acceptance**	23	26028	39567	62.6% (55.6–69.4)	3397.3	99.4%	<0.0001
**Gender**							
**Male**	12	7646	32294	33.3% (26.1–41.0)	1462.4	99.2%	<0.0001
**Female**	13	8697	33262	35.7% (29.4–42.3)	1232.0	99.0%	<0.0001
**Residence**							
**Rural**	5	1592	7781	20.14%(16.8–23.7)	54	92.6%	<0.0001
**Urban**	5	2478	7781	30.7% (23.0–39.0)	225	98.2%	<0.0001
**Semiurban**	3	454	2887	11.7%(2.10–27.50)	236.8	99.2%	<0.0001

## Data Availability

Data supporting this systematic review is available in the reference section.

## Supplementary Materials

The following supporting information can be downloaded at: https://www.mdpi.com/article/10.3390/vaccines11050964/s1, File S1: PRISMA Checklist 2020.
